# Hypothyroidism Reduces the Size of Ovarian Follicles and Promotes Hypertrophy of Periovarian Fat with Infiltration of Macrophages in Adult Rabbits

**DOI:** 10.1155/2017/3795950

**Published:** 2017-01-04

**Authors:** J. Rodríguez-Castelán, M. Méndez-Tepepa, Y. Carrillo-Portillo, A. Anaya-Hernández, J. Rodríguez-Antolín, E. Zambrano, F. Castelán, E. Cuevas-Romero

**Affiliations:** ^1^Centro Tlaxcala de Biología de la Conducta, Universidad Autónoma de Tlaxcala, Tlaxcala, TLAX, Mexico; ^2^Departamento de Biología de la Reproducción, Instituto Nacional de Ciencias Médicas y Nutrición Salvador Zubirán, Mexico City, Mexico; ^3^Departamento de Biología Celular y Fisiología, Instituto de Investigaciones Biomédicas, Unidad Periférica Tlaxcala, UNAM, Ciudad de México, Mexico

## Abstract

Ovarian failure is related to dyslipidemias and inflammation, as well as to hypertrophy and dysfunction of the visceral adipose tissue (VAT). Although hypothyroidism has been associated with obesity, dyslipidemias, and inflammation in humans and animals, its influence on the characteristics of ovarian follicles in adulthood is scarcely known. Control and hypothyroid rabbits were used to analyze the ovarian follicles, expression of aromatase in the ovary, serum concentration of lipids, leptin, and uric acid, size of adipocytes, and infiltration of macrophages in the periovarian VAT. Hypothyroidism did not affect the percentage of functional or atretic follicles. However, it reduced the size of primary, secondary, and tertiary follicles considered as large and the expression of aromatase in the ovary. This effect was associated with high serum concentrations of total cholesterol and low-density lipoprotein cholesterol (LDL-C). In addition, hypothyroidism induced hypertrophy of adipocytes and a major infiltration of CD68+ macrophages into the periovarian VAT. Our results suggest that the reduced size of ovarian follicles promoted by hypothyroidism could be associated with dyslipidemias, hypertrophy, and inflammation of the periovarian VAT. Present findings may be useful to understand the influence of hypothyroidism in the ovary function in adulthood.

## 1. Introduction

Obesity, dyslipidemias, and inflammation markers are common findings in women with ovarian infertility [1, 2]. Furthermore, hypertrophy and dysfunction of the visceral adipose tissue (VAT) have been associated with ovarian failure [3–5], suggesting an influence of periovarian environment in the follicles maturation. Other studies conducted in domestic and laboratory animals have confirmed that alterations in metabolism affect the ovarian function [6–9].

There is an important occurrence of infertility and anovulation in hypothyroid patients [10, 11], which may involve metabolic alterations and inflammation [12, 13]. However, scarce studies have approached the influence of thyroid dysfunction in the ovarian failure. In adult rats, the effect of hypothyroidism on the ovarian function is confused depending on the estrous cycle phase [14]. Further studies are necessary to analyze the influence of hypothyroidism in the maturation of follicles in adulthood.

The present study therefore aimed to analyze the influence of hypothyroidism in the number and size of ovarian follicles and the aromatase expression in the ovary of adult rabbits. We also investigated the possible association between ovary changes, metabolic indicators, and hypertrophy of periovarian VAT.

## 2. Methods

### 2.1. Animals and Treatments

Twelve Chinchilla-breed virgin female rabbits* (Oryctolagus cuniculus)* were housed under controlled temperature (20 ± 2°C) and light : dark cycle of 16 : 8 h. By using these conditions, most of females are in an early proestrus phase [15]. They were daily provided with pellet food (120 g/day) and tap water ad libitum. Hypothyroidism was induced by the administration of 0.02% methimazole (Sigma; approximate diary dosage 10 mg/kg) in drinking water for one month. This dose reduces serum concentrations of thyroid hormones and increases concentrations of thyrotropin (TSH) in rabbits as previously reported [15]. At the end of this treatment, control and hypothyroid rabbits were anesthetized with sodium pentobarbital (60 mg/kg, i.p.) and subsequently euthanized with an overdose of the same anesthetic. The Ethics Committee from the Universidad Autónoma de Tlaxcala, according to the guidelines of the Mexican Law for Production, Care, and Use of Laboratory Animals, approved this experimental design.

### 2.2. Ovary Follicles Analysis

Immediately after death, left ovaries were excised, fixed in Bouin-Duboscq fixative, dehydrated, and embedded in paraplast X-tra (Sigma-Aldrich). Afterwards, tissue was longitudinally cut at 7 *μ*m using a microtome (Thermo Scientific, Model 325). Tissue sections were mounted on gelatin-coated slides (Sigma-Aldrich). Three sections (two laterals and one from the middle portion, separated by 2000 *μ*m) were selected. These were deparaffinized, rehydrated, and stained with Masson trichrome stain. Pictures were taken at 4x with an optical microscope (Axio Imager A1, Zeiss) equipped with an Olympus digital camera with a resolution of 5.1 megapixels, and a complete reconstruction of each ovarian section was done. The cross-sectional area (CSA) of each ovarian section was measured by using the programs Axiovision Release 4.8 (Zeiss Software Inc.) and ImageJ 1.43 (National Institutes of Health, Bethesda, MD). The numbers of healthy primordial, primary, secondary, and tertiary follicles were counted in the three ovary sections per rabbit. Follicles that exhibited an organized granulosa cell layer were considered as healthy follicles. They were classified as follows [16]: primordial follicle (an oocyte surrounded by one layer of flattened granulosa cells), primary follicle (an oocyte surrounded by one layer of cuboidal granulosa cells), secondary follicle (diverse layers of cuboidal granulosa cells without antral space), tertiary follicle (diverse layers of cuboidal granulosa cells with antral space), and Graafian follicles (presence of cumulus and granulosa and theca cell layers). The numbers of atretic follicles grade I (cystic and invasive atresia) and grade II (obliterative and residual atresia) were also counted [17]. The cystic atresia is characterized by a disorganization of granulosa and theca cells (some granulose cells invade the antrum). The invasive atresia or atresia associated with luteinization is characterized by hypertrophy of granulosa and theca cells, which leads to a local rupture of the basal membrane and to a disturbance of regular layout of granulosa cells. Obliterative or collapsing atresia was identified by the degeneration of granulosa cell layer and presence of fibrotic connective tissue. Residual atresia showed oocyte and pellucid zone residues. No granulosa and theca cells were observed.

In the three images of reconstructed ovarian sections (see above), a randomized selection of quadrants was done to measure the CSA of primordial, primary, secondary, and tertiary follicles, using the Axiovision program. Graafian follicles were not analyzed due to their low number. From the CSA, the diameters of follicles were obtained.

### 2.3. Aromatase Expression in the Ovary

The expression of aromatase in the ovary was done by Western blot as reported elsewhere [18]. Portions (anterior, middle, and posterior) of right ovaries from each rabbit were used to get 50 mg of tissue and to obtain total protein extracts. Thirty micrograms of total protein, approximately, were resolved onto SDS-PAGE and electroblotted to nitrocellulose membranes (Bio-Rad Laboratories Headquarters). Membranes were stained with Ponceau's Red to confirm that protein content was equal in all lines. Membranes were soaked in 17.0% milk in tris-saline buffer (TSBT) containing 0.2% tween and incubated overnight at 4°C with an affinity purified polyclonal antibody anti-P450 aromatase (1 : 200, NB100-1596, Novus Biologicals) followed by secondary antibodies (1 : 20000, goat anti-rabbit IgG, sc-2004, Santa Cruz Biotechnology). Immunoreactive polypeptides were detected using a chemiluminescence kit (West Pico Signal, Thermo Scientific). Chemiluminescent signal was captured and analyzed with a chemiluminescent-signal analyzer (MyECL Imager, Thermo Fisher Scientific). The expression of P450 aromatase was measured by densitometry and normalized against the signal obtained from Ponceau's Red staining used as loaded control [19]. For this, the ImageJ software (National Institutes of Health, USA) was used. Data are the quotient (arbitrary units, a.u.) obtained by dividing the density of the aromatase band by the density of bands covering at least 90% of the length of each lane as seen after Ponceau's Red staining [19]. This was done for each ovary.

The distribution of aromatase in the different types of follicles was approached by immunohistochemistry. Some slides were deparaffinized and incubated in microwave-heated 10 mM sodium citrate pH 6 to retrieve antigens. Endogenous peroxidases were quenched with 0.3% hydrogen peroxide diluted in phosphate buffer saline (PBS). Endogenous binding sites for secondary antibodies were blocked with 10% normal goat serum diluted in PBS with 0.3% Triton X-100 (PSBT). Anti-P450 aromatase (1 : 1000, NB100-1596, Novus Biologicals; for 24 h at 4°C) and the secondary antibody (1 : 2000, goat anti-rabbit IgG; for 2 h at 37°C) were used. Immunostaining was developed according to the Vectastain ABC kit directions (Vector Labs), using 0.05% 1,3′-diaminobenzidine (Sigma-Aldrich) and 0.01% H_2_O_2_ as enzyme substrate. Sections were washed and counterstained with Mayer's hematoxylin. Sections were rinsed, dehydrated in ethanol, cleared in xylene, and mounted. Sections were photographed.

### 2.4. Metabolic Variables

Blood samples were obtained from cardiac puncture of anesthetized female rabbits that had a fastening of 12 h. Serum was stored at −80°C until assayed. Total cholesterol and triacylglycerol (TAG) levels were measured using standard enzymatic methods (ELITech, France). The high-density lipoprotein cholesterol (HDL-C) was measured by a precipitating method (ELITech, France). The concentration of low and very low-density lipoprotein (LDL-C and VLDL-C) was calculated from the concentration of TAG, by using the Friedewald equations: [_VLDL-C_] = 0.2 × [TAG], and [_LDL-_C] = [_TOTAL_C] − [C_HDL-C_] − [_VLDL-C_]. Serum uric acid was measured using an automatic photometry method that was carried out by a commercial laboratory (CARPERMOR Laboratories, S.A. de C.V.). Serum leptin concentrations were measured by RIA using commercial kits obtained from Linco Research, Inc. (Cat. number RL-83K) [20].

### 2.5. Periovarian VAT Analysis

Visceral fat around ovaries was excised and histologically processed as described in the ovarian follicles analysis subsection. One slide per rabbit was stained with the hematoxylin-eosin staining. Pictures at 40x were taken from 6–8 microscopic fields and CSA of 40–50 adipocytes was measured using the program Axiovision. The average of the CSA was obtained.

Other slides were deparaffinized and processed for immunohistochemistry as aforementioned. Endogenous peroxidases were quenched and endogenous binding was blocked with 5% of goat or rabbit serum. Independent sections were incubated with primary antibodies to detect macrophages immunoreactive to CD68 (1 : 200, mouse monoclonal antibody Santa Cruz Biotechnology for 24 h at 4°C) [21] or CD163 (1 : 200, goat polyclonal antibody Santa Cruz Biotechnology for 72 h at 4°C) [21]. Subsequently, they were incubated with secondary antibodies (1 : 250; goat anti-mouse IgG or donkey anti-goat, resp.) and diluted in PBST for 2 h at 37°C. Immunostaining was developed using the ABC method and sections were washed and counterstained with Mayer's hematoxylin. Pancreas of rabbits was used to test the specificity of the anti-CD68 antibody [22]. Likewise, the immunolabeling of periovarian VAT with the goat polyclonal to perilipin A (1 : 100; Abcam Inc., ab60269) was assayed to test the specificity of the anti-goat secondary antibody. Nonspecific immunostaining was observed in adipose tissue when primary antiserums were omitted. Sections were photographed at 100x; ten microscopic fields were used to count the number of CD68 or CD163 positive macrophages. In addition, the number of immune cells by field was counted in 20 microscopic fields.

### 2.6. Statistical Analyses

The statistical analysis was performed with the program GraphPad Prism v 5.01 (GraphPad Software, Inc., CA, USA). The CSA of all types of follicles was adjusted to two standard deviations to discard out layers. Results were expressed as mean ± SEM for each variable. Kolmogorov-Smirnov tests were used to analyze the normality of data. Student's* t-* or Mann-Whitney* U* tests were used to determine significant differences between control and hypothyroid rabbits, considering the normality of the data for each comparison. A value of *P* ≤ 0.05 was considered statistically significant.

## 3. Results

When female rabbits were sacrificed, age (11.8 ± 0.2 months old for control and 11.2 ± 1.2 months old for hypothyroid animals) and body weight (4.2 ± 0.1 kg for control and 4.1 ± 0.1 kg for hypothyroid animals) were similar between groups.

**Figure 1:**
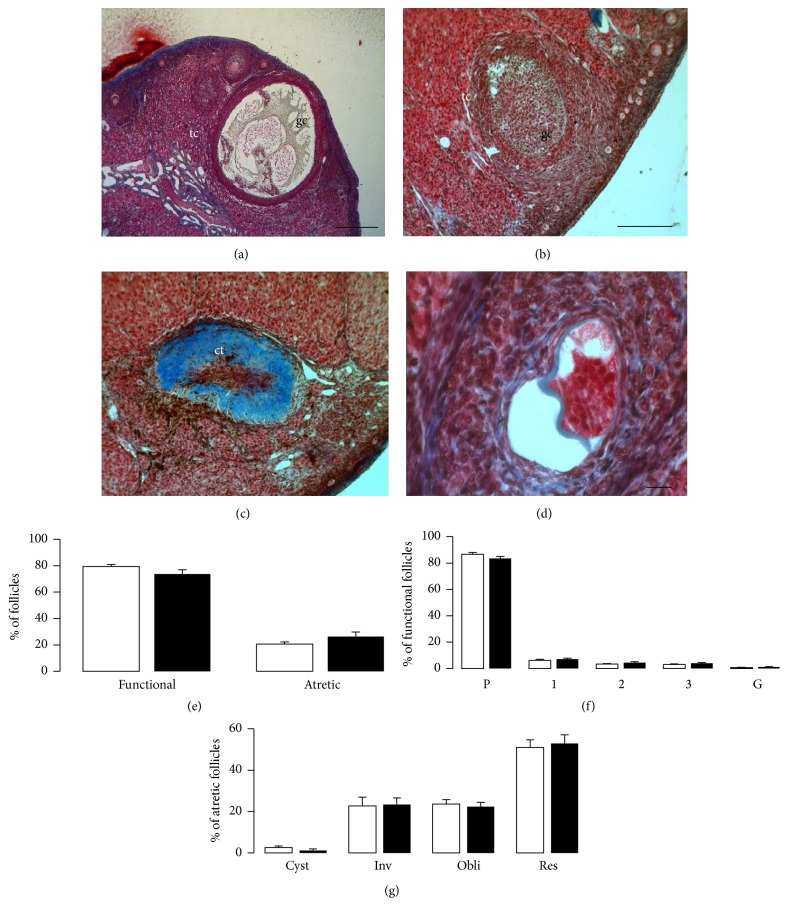
Follicle morphometry in control and hypothyroid rabbits. Atretic follicles: (a) cystic (Cyst), (b) invasive (Inv), (c) obliterative (Obli), and (d) residual (Res). Bars: (a) 500 *μ*m, (b-c) 200 *μ*m, and (d) 20 *μ*m. Granulose cells, gc; theca cells, tc; and connective tissue, ct. (e) Percentage of ovarian functional and atretic follicles of control (open bars; *n* = 6) and hypothyroid (solid bars; *n* = 6) females rabbits. (f) Functional follicles were classified as primordial (P), primary (1), secondary (2), tertiary (3), and Graafian (G). (g) For atretic follicles, the different type of atresia mentioned above was analyzed. Data are mean ± SEM.

Considering the three reconstructed ovarian sections (*see* Methods), the means of the CSA of the ovary were similar between groups (13243.8 ± 1532.9 mm^2^ for the control group and 11180.8 ± 2040.3 mm^2^ for the hypothyroid group). A mean of 317.8 ± 30.9 of both functional and atretic follicles for the control group and 352.2 ± 44.7 follicles for the hypothyroid group were counted. For both groups, 80% were functional follicles and 20% were atretic (Figures 1(a)–1(d)). No differences in the percentages of functional and atretic follicles between groups (Figure 1(e)) or in the percentage of primordial, primary, secondary, and tertiary follicles (Figure 1(f)) were found. Likewise, the percentages of the different types of atretic follicles (cystic, invasive, obliterative, and residual) were similar between groups (Figure 1(g)).

**Figure 2:**
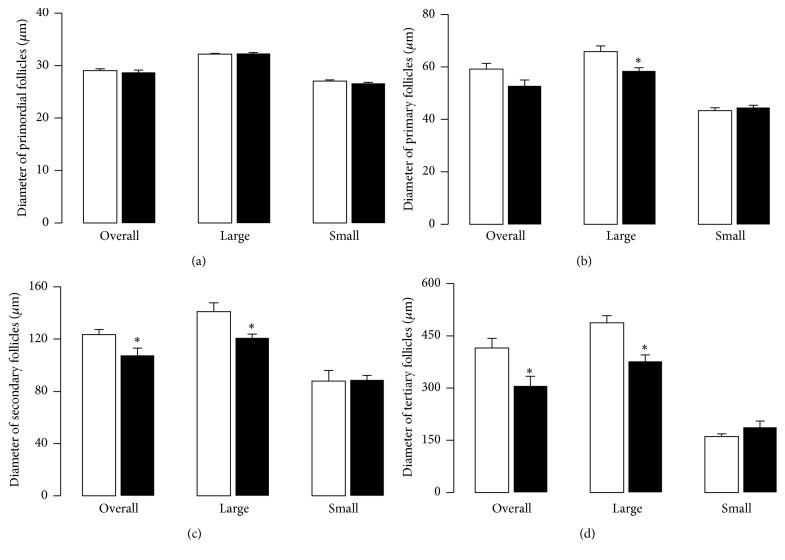
Diameter of primordial (a), primary (b), secondary (c), and tertiary (d) ovarian follicles of control (open bars; *n* = 6) and hypothyroid (solid bars; *n* = 6) females rabbits. The mean diameter for all (overall), large, and small follicles was analyzed. Large follicles were considered: primordial > 30 *μ*m, primary > 50 *μ*m, secondary > 100 *μ*m, and tertiary > 250 *μ*m. Data are mean ± SEM. ^*∗*^*P* ≤ 0.05.

The randomized selection of functional follicles in the three reconstructed ovarian sections (*see* Methods) permitted the measurement of the CSA of 207 primordial, 60 primary, 25 secondary, and 37 tertiary follicles for the control group. Meanwhile 196 primordial, 75 primaries, 37 secondary, and 39 tertiaries were analyzed for the hypothyroid group. The means of diameters for primordial and primary follicles were similar between groups (Figures 2(a)-2(b)). In comparison with the control group, low averages of diameters for the secondary and tertiary follicles for the hypothyroid group were found (Figures 2(c)-2(d)). By considering the frequency of distribution of follicle size in control rabbits, follicles were classified as large and small. Large follicles were considered as primordial > 30 *μ*m, primary > 50 *μ*m, secondary > 100 *μ*m, and tertiary > 250 *μ*m. This arbitrary classification of follicles by size showed that the percentages of large primordial (37.8 ± 3.8 versus 37.4 ± 6.9), primary (66.3 ± 8.6 versus 55.8 ± 9.7), secondary (62.5 ± 8.5 versus 55.4 ± 12.5), and tertiary (71.3 ± 9.0 versus 55.5 ± 14.9) follicles between the control and hypothyroid groups, respectively, were similar. This was also true for the small follicles (data are not shown because they are complementary to large ones). For its part, the diameter of large and small primordial follicles, respectively, was similar between groups (Figure 2(a)). However, the diameter of large primary, secondary, and tertiary follicles was smaller for the hypothyroid group (Figures 2(b)–2(d)).

**Figure 3:**
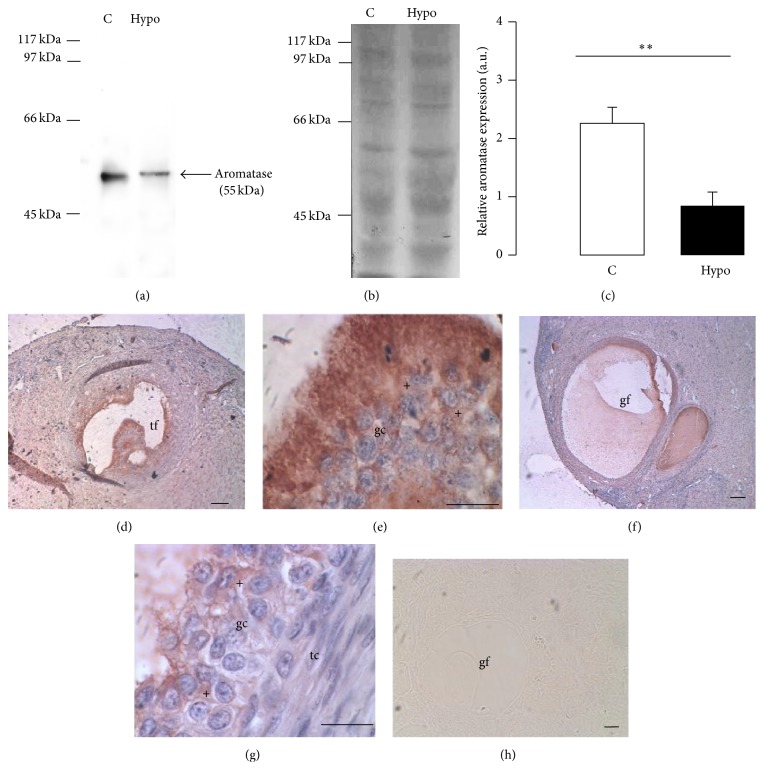
Expression of the cytochrome P450 aromatase in the ovary of control (C; open bar; *n* = 6) and hypothyroid (Hypo; solid bar; *n* = 6) rabbits. Representative immunoblot showing the expression of aromatase (a) and Ponceau's Red stained membrane (b). Relative expression of aromatase in C and Hypo groups (c). Data are mean ± SEM. ^*∗∗*^*P* < 0.01. Immunoreactivity of antiaromatase (+) was identified in granulosa cells of antral follicles (tertiary, tf; Graafian, gf) of control (d, e) and hypothyroid (f, g) ovaries. Nonlabeling was observed when the primary antibody was omitted (negative control; h). Bars: (d) 200 *μ*m; (f) and (h) 100 *μ*m; and (e) and (g) 20 *μ*m. Granulose cells, gc; theca cells, tc.

Furthermore, the concentration of aromatase in the ovary for the hypothyroid group was lower than that for the control group (Figures 3(a)–3(c)). Immunohistochemistry revealed that aromatase was only expressed in the granulosa cells of antral follicles. No immunoreactivity was detected in the granulosa cells of preantral follicles. In spite of the fact that aromatase immunoreactivity was not quantitated, the intensity of labeling in antral follicles from hypothyroid ovaries was weaker than in those from control ones (Figures 3(d)–3(h)).

The serum concentrations of total cholesterol and LDL-C for hypothyroid females were higher than those for control ones (Table 1). The concentrations of TAG, HDL-C, VLDL, uric acid, and leptin were similar between groups (Table 1).

**Figure 4:**
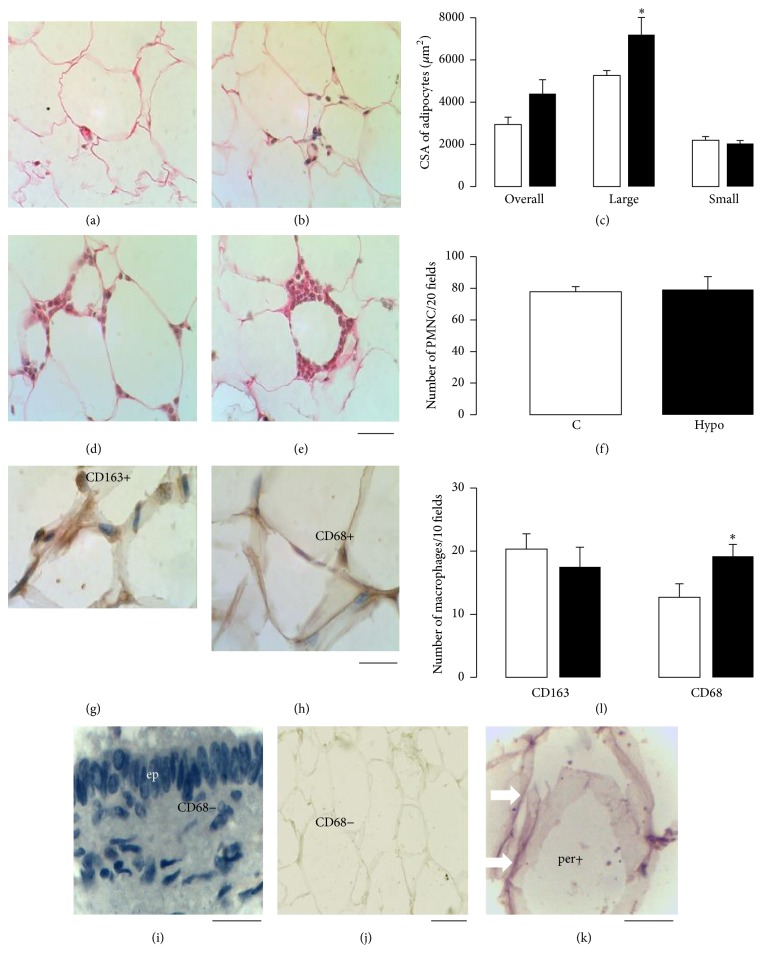
Histological characteristics of periovarian adipocytes of control (a and d) and hypothyroid (b and e) females rabbits. (c) The cross-sectional area of adipocytes (CSA; control in open bars; *n* = 6; hypothyroid in solid bars; *n* = 6). The average CSA for all (overall), large, and small adipocytes was analyzed. Large adipocytes were considered ≥4000 *μ*m^2^. The presence of immune cells (polymorphonuclear cells; PMNC) around adipocytes was quantified (f). Data are mean ± SEM. ^*∗*^*P* ≤ 0.05. Immunoreactivity of macrophages to CD163 and CD68 markers was analyzed (g, h, and l). The epithelium (ep) of the pancreatic duct in rabbits was considered as a negative control for anti-CD68 (i). The lack of the primary antibody (j) and the immunohistochemistry antiperilipin A (white arrows per+) in periovarian adipocytes were considered as negative controls for the secondary antibodies anti-mouse and anti-goat, respectively. Bars: (a), (b), (d), (e), and (j) = 50 *μ*m; (g), (h), (i), and (k) = 20 *μ*m.

Hypothyroid females had hypertrophied periovarian adipocytes (Figures 4(a)-4(b)). The CSA of 239 adipocytes for the control group and 271 adipocytes for the hypothyroid group were measured. The average of the CSA of adipocytes was similar between groups (Figure 4(c)). For each group, adipocytes were arbitrarily classified as large (≥4000 *μ*m^2^) and small (<4000 *μ*m^2^) in accordance with the frequency distribution of their CSA. By doing this, it was noted that the size of large adipocytes for the hypothyroid group was higher than those for the control group (Figure 4(c)). The present crown-like structures surrounding adipocytes were observed in both groups (Figures 4(d)-4(e)), and the number of immune cells was similar between groups (Figure 4(f)). Some macrophages were positive to CD163 and CD68 (Figures 4(g)-4(h)). The epithelium of the pancreatic duct in rabbits was considered as a negative control for anti-CD68 (Figure 4(i)). The lack of the primary antibody (Figure 4(j)) and the immunohistochemistry for antiperilipin A (Figure 4(k)) in periovarian adipocytes were considered as negative controls for the secondary antibodies anti-mouse and anti-goat, respectively. The number of CD163+ macrophages was similar between groups, but a higher number of CD68+ macrophages in hypothyroid rabbits were found (Figure 4(l)).

## 4. Discussion

Hypothyroidism did not affect the number of functional or atretic follicles of adult rabbits. However, it reduced the size of large primary, secondary, and tertiary follicles. In agreement with studies conducted in pigs and rodents, our finding could be related to an inadequate quality of follicles [23, 24]. Furthermore, the reduced size of largest preantral and antral follicles could be related to a failure involving the gonadotropins or/and growth factor signaling, as well as capillary networks alterations [25].

Hypothyroidism also affected the expression of aromatase in the ovary, which was low compared to the control group. Certainly, this expression represents only a portion of the ovary and the type or number of follicles present is unknown. The reduced expression of aromatase agrees with other studies done in pigs and humans, in which small follicles have a minor expression of aromatase accompanied by a less concentration of estradiol in the follicular fluid [26, 27]. Moreover, immunohistochemical labeling showed that aromatase was only expressed in the granulosa cells of antral follicles. This finding agrees with another study done in mares in which the immunostaining antiaromatase in the granulose cells depends on the size and morphology of the follicle supporting the aromatase expression is higher in large preovulatory follicles than in small ones [28]. Similar results have been reported in goats using the RT-PCR technique [29]. Our present results therefore suggest that the low expression of aromatase in the ovary could be related to the small size of large tertiary follicles (antral follicles). Likewise, this low aromatase expression in antral follicles of hypothyroid animals could be related to modifications in the formation and function of the corpus luteum [30], rather than changes in the folliculogenesis [31]. In this regard, hypothyroidism affects the proliferation, angiogenesis, and apoptosis in the corpus luteum of rats [32].

Considering that granulose and theca cells of primary, secondary, and tertiary follicles, as well as the stroma of the rabbit, express thyroid hormone receptors (TRs) and TSH receptors (TSHR) [33], a direct effect of thyroid hormones and TSH on this tissue could be assumed. In contrast to those studies in humans and animals in which hypothyroidism promotes the formation of cystic follicles [34, 35], we did not find more cystic follicles in hypothyroid rabbits. However, it is important to take into account that rabbits are reflex ovulators [36] and females used in this study were at early proestrus [15]. For this, the presence of luteal cysts should be analyzed in the luteal phase [37].

Hypothyroid rabbits had higher serum concentrations of cholesterol and LDL-C than control females. Our results agree with other studies done in rats and mares [38, 39] confirming the link between hypothyroidism and dyslipidemias. Considering that there is a positive correlation between the concentrations of total cholesterol in serum and in the follicular fluid [40], and that cholesterol is useful to the maturation and grown of follicles [40], hypercholesterolemia may be involved in the reduction of follicle size reported herein. Further studies are necessary to test whether hypothyroidism could modify the amount of cholesterol in the ovarian follicles depending on their size as has been reported in sheep and cattle [40, 41]. Other metabolic indicators like TAG, uric acid, and leptin were normal in hypothyroid rabbits. In contrast, some studies done in rats or mares with hypothyroidism induced by propylthiouracil or thyroidectomy, respectively [38, 39], have reported an increase in the serum concentrations of TAG, VLDL, and HDL-C. These discrepancies could be related to the animal species and/or to the method of induction of hypothyroidism used. For its part, the effect of hypothyroidism on the serum concentration of leptin in hypothyroid animals is confused because some studies have showed a decrease in the circulating leptin of hypothyroid rats [42], while others have not [43]. Otherwise, adiposity and ovarian dysfunction have been associated with hyperuricemia, which has been used as a metabolic marker [44] related to hyperandrogenism [1]. In this regard, hypothyroid rabbits had normal serum concentrations of uric acid, but they also have normal serum concentrations of testosterone [15]. However, a modification of these last metabolic markers in the ovarian environment should not be discarded because the presence of TAG and leptin in the follicular fluid has been associated with the number and size of follicles in cows and pigs [40, 45]. Indeed, uric acid can act as antioxidant, which could affect the follicle size [46].

Hypothyroid females also showed a hypertrophy of periovarian VAT adipocytes. This finding could be related to an increment in the TAG stored and a decrease in the TAG lipolysis promoted by hypothyroidism [47]. In this regard, adipocytes from the VAT of diverse mammals express TRs and deiodinases, suggesting a direct effect of thyroid hormones on this tissue [48]. Additionally, dyslipidemias and hypertrophy of the periovarian VAT found in hypothyroid virgin rabbits seems to be accompanied by steatohepatitis [49]. This conjunction of results could help to explain the link between ovarian dysfunction and nonalcoholic fatty liver disease, obesity, insulin resistance, and dyslipidemias observed in women [50]. In general, hypertrophy of adipocytes from the VAT has been associated with an increase in the infiltration of macrophages that can alter the adipocyte function [51]. Our findings agree with other studies reporting that thyroid hormones regulate the infiltration of macrophages into the peritoneal cavity and their activation in rodents [52, 53]. Furthermore, hypothyroidism favors the adipokine synthesis and signaling in the white adipose tissue of rats [54].

Several reports support direct actions of macrophages in healthy and atretic follicles, as well as in the corpora lutea, participating in the apoptosis, angiogenesis, maturation, and steroidogenesis [55, 56]. However, few studies have suggested that a major infiltration of macrophages in the visceral, periovarian, or subcutaneous adipocytes may also be involved in the maturation of follicles in cows and mice [57, 58]. Our present findings show an important presence of CD68+ macrophages into the periovarian VAT of hypothyroid rabbits. This may be associated with a small size of ovarian follicles. Considering that the CD68 glycoprotein modulates the phagocytic activity of macrophages in the ovary [59], it is possible to suggest that the increment in the number of macrophages in the periovarian VAT could be related to plausible ovarian follicles damage in hypothyroid rabbits that could affect their size.

In conclusion, our present study shows that the reduced size of primary, secondary, and tertiary follicles and the reduced ovarian aromatization found in hypothyroid rabbits could be associated with an increment in the serum total cholesterol, as well as with a hypertrophy and dysfunction of the periovarian VAT. Present findings may be useful to understand the association between hypothyroidism and ovarian infertility.

## Figures and Tables

**Table 1:** Metabolic variables from control and hypothyroid female rabbits. ns, nonsignificant. Data are mean ± SEM. ^a^It was measured in 5 rabbits.

Variable	Control group *n* = 6	Hypothyroid group *n* = 6	Statistics
Total cholesterol (mg/dL)	64.0 ± 5.8	92.3 ± 5.0	*t* = 3.6; *P* < 0.004
Triacylglycerol (mg/dL)	70.3 ± 8.4	66.0 ± 10.4	ns
LDL-C (mg/dL)	10.4 ± 5.6	32.8 ± 5.5	*t* = 2.8; *P* < 0.01
HDL-C (mg/dL)	39.5 ± 1.1	46.3 ± 3.5	ns
VLDL (mg/dL)	14.1 ± 1.7	13.2 ± 2.1	ns
Uric acid (mg/dL)	0.6 ± 0.1	0.7 ± 0.1	ns
Leptin (ng/mL)	1.5 ± 0.2	^a^1.3 ± 0.1	ns
